# A Predictive Model for USMLE Step 1 Scores

**DOI:** 10.7759/cureus.769

**Published:** 2016-09-07

**Authors:** Christin Giordano, David Hutchinson, Richard Peppler

**Affiliations:** 1 Faculty and Academic Affairs, University of Central Florida College of Medicine

**Keywords:** step 1, usmle, step 1 score

## Abstract

**Purpose:**

The United States Medical Licensing Examination (USMLE) Step 1 plays a pivotal role in one’s residency application. While prior literature has investigated which factors influence performance on the examination, the authors sought to include factors such as performance on a well-used question bank and financial need to develop a predictive model.

**Method:**

After obtaining institutional review board approval, the authors surveyed two consecutive second-year medical school classes and correlated the data to the students’ Step 1 and National Board of Medical Examiners (NBME) Comprehensive Basic Science Examination (CBSE) scores. The survey included questions such as how many days they studied per week, how many days they studied in total, which resources they used, how they performed on question banks, group studying habits, and whether they were receiving financial aid. The authors also assessed whether the students received only A letter grades during the first year of medical school. The authors used SPSS® Statistics V22.0 (IBM® Corporation, NY, USA ) and included one-way analysis of covariance (ANOVA) and multiple linear regression for statistical analysis.

**Results:**

Eighty-one students completed the survey with an average Step 1 score of 240.5 and with an average study time of 39.5 days. The Step 1 Scores significantly correlated with the CBSE taken immediately preceding the dedicated study period (r=0.711, *P*=<0.001), UWorld Question Bank (UWorld) percentage correct (r = 0.622, *P*<0.001), straight As during first-year (r=0.356, *P*=0.001), and financial need (r=0.318, *P*=0.01). The scores were not correlated with age, gender, Medical College Admissions Test (MCAT), prior medical training, number of days studied, or the students’ perception of appropriate time studied. The authors developed a predictive model accounting for 62.3% of the variability. 140.625+(0.319xCBSE)-(3.817xA)+(5.845xN)+(0.452xU), where A=1 if straight As, N=1 if receiving need-based scholarship, U=UWorld percent-correct, and CBSE=the three-digit score of the CBSE taken prior to the dedicated study period.

**Conclusions:**

Academic performance and financial need may predict Step 1 scores. Interestingly, the number of days studied did not have a correlation with scores, suggesting that increased length of study may not ameliorate poor grades.

## Introduction

The United States Medical Licensing Examination (USMLE) Step 1 exam is the first of three required exams for the practice of medicine as a physician within the United States. Because studies have found that higher USMLE scores are associated with improved faculty evaluations and first-time board pass rates, it is held in extremely high regard by residency programs nationwide and can affect placement into competitive residencies [1-2]. In fact, in the 2014 National Resident Matching Program Director Survey, 94% of the 1,793 residency programs directors spanning all medical specialties cited Step 1 scores as an important factor with a rating of 4.1 out of 5, and it was cited more than any other factor in the survey [3]. As such, the Step 1 exam is arguably the most pivotal assessment undertaken in medical school and thus medical students approach the exam very seriously.

In order to succeed on the USMLE Step 1 exam, students employ a wide variety of study habits and utilize an array of study resources. Naturally, study habits and ability are unique to each individual but one can attempt to observe overall patterns. In their preparation, many students use specific study aids such as First Aid for the USMLE Step 1 and Pathoma, which offer rapid review and exposure to relevant content that may appear on the exam. Most students also utilize question banks such as UWorld Question Bank (UWorld) or Kaplan QBank to familiarize themselves with USMLE-style questions and content. These question banks in combination with practice exam materials such as the National Board of Medical Examiners (NBME) Comprehensive Basic Science Examination (CBSE), often give students an approximate idea of their level of knowledge and how much preparation is still required [4].

Outside of study resources, other variables may also play significant roles in exam performance. Whether or not the student had a science background before attending medical school may influence their understanding of certain concepts. A surrogate measurement for prior scientific knowledge may be performance on the Medical College Admission Test (MCAT), particularly on the biological and physical sciences sections. In fact, Julian performed a prospective study of two cohorts of medical school classes to examine the use of undergraduate grade point average (GPA) and MCAT to predict performance in medical school and beyond. He found that the contribution of undergraduate GPA to performance on Step 1 was overtaken by performance on the MCAT, and thus MCAT scores could be used as a surrogate measure of undergraduate performance. He found that there was an overall pattern of better academic performance associated with higher MCAT scores, in all sections of the exam [5]. Further, Veloski, et al. performed a retrospective study on 6,239 students entering medical school over 30 years and correlated MCAT scores, undergraduate GPA, age, and sex to performance on the Step 1 examination. These authors found that for each point increase in the MCAT science score, there was a 4.26-point increase in the Step 1 score [6]. These findings were consistent with other studies, which have demonstrated a correlation of MCAT scores to Step 1 scores [7-8].

While MCAT performance may demonstrate knowledge prior to entering medical school, most of the information tested on Step 1 is related to information learned in the first two years of medical school. Therefore, it would not be a stretch to think that students who perform well during these two years would have better performance on the examination. In fact, Johnson, et al. found that performance on exams throughout the first- and second-year curriculums were highly correlated to scores on the Step 1 exams. Further, correlations were particularly high for those exams that had more components of physiology and/or pathophysiology content [9].

Step 1 examination preparation, is for most people, a daunting prospect. Tutoring or peer support to provide structure and improve understanding of important concepts may aid in student preparation. In fact, Alcamo, et al. examined how a peer-led Step 1 review course may affect Step 1 scores. They surveyed students following participation and correlated the students’ exam scores to participation in the review course. Interestingly, the participants had a mean Step 1 score approximately eight points higher than nonparticipants (*P*=0.005) [10]. However, we were unable to find a study in current literature that examines how students independently study or the number of days during the dedicated study period often afforded to students preparing for the Step 1 examination. We were also unable to find literature on which study resources students typically use during their preparation.

In our study, we sought to determine how students at our institution prepared for the examination, and we included factors like the most popular resources, group study habits, and prior tutoring experience. Using prior studies as a guide, we developed a hypothesis. We hypothesized that students who excelled in their first and second year of medical school curriculum will outperform their peers when other factors are controlled. We also anticipated that those students who used supplemental question banks, such as UWorld, would also achieve higher Step 1 scores. Lastly, we believed we could develop a predictive model based on academic performance during medical schools, CBSE performance, UWorld performance, number of days studied during a dedicated study period, financial need, and MCAT score.

## Materials and methods

At our institution, the Step 1 examination is taken during a dedicated study period, which lasts a total of seven weeks. At the start of this period, the students are required to take a school-issued National Board of Medical Examiners (NBME) Comprehensive Basic Science examination (CBSE) after which the students may study on their own for the remainder of the time. The student may take the Step 1 examination at any point during this period but must take it before the end of the period, at which point the third-year orientation is held. The authors designed a survey that asked the students questions about how they prepared for the Step 1 examination and their feelings of overall preparedness. The questions included items such as how many days they studied per week, how many days they studied in total, which resources they used, how they performed on question banks they used (given in percentage), whether they participated in group study, and whether they were receiving financial aid in the form of a need-based scholarship. Also included was a Likert-scale type item on how prepared the students felt for the examination. Lastly, we asked whether they had been selected as a Peer Academic Coach (PAC), i.e. a student who is selected to coach or tutor their peers. PACs must have received only A letter grades during the first year of medical school, thus we used PAC designation as a surrogate for academic performance during the first-year of medical school.

In two consecutive years, we used an electronic survey tool, Qualtrics (Qualtrics, LLC, Utah, USA), to disseminate the survey to students during third-year orientation, prior to receiving their Step 1 scores. The survey was emailed to all eligible rising third-year students in two consecutive classes (n=213). Other variables used for this study included individual scores on the United States Medical Licensing Examination (USMLE) Step 1 examination, CBSE, and performance on the Medical College Admissions Test (MCAT), all of which the Office for Planning and Knowledge Management supplied to the authors in a deidentified manner. The University of Central Florida Institutional Review Board reviewed and approved the study protocol.

We analyzed data and generated descriptive statistics using SPSS® Statistics V22.0. We matched individual students’ Step 1 scores, CBSE scores, and total MCAT scores to their survey responses that were deidentified and returned to the authors for analysis, and we treated Likert-scale items as ordinal data. We determined any correlations with Pearson’s correlation coefficient between Step 1 scores and variables such as financial need, number of hours a student tutored others, and days studied. We used independent-samples t-test to compare the mean Step 1 scores of those who group studied and those who had not, to compare the mean scores of those who were PACs and those who were not, and to compare the mean scores of those receiving financial aid to those who were not. We used one-way analysis of variance (ANOVA) to compare mean Step 1 scores between three groups of PACs separated by how many reported hours they worked per month and five groups of students divided by how many days they studied. We developed a predictive model utilizing stepwise multiple linear regression analysis with the assumption that our dependent variable, Step 1 scores, was measured on a continuous scale and that our independent variables can be measured on continuous or categorical scales. Our independent variables were the following: whether or not a student received straight As during the first year, financial need, CBSE score, and percentage correct on the UWorld question bank. Further, we used the Durbin-Watson statistic to confirm that there was independence of observations. Lastly, we generated a histogram with a normal probability plot to ensure that our residuals were normally distributed. We considered data statistically significant if *P*<0.05.

## Results

The survey response rate was 38.5% (82/213), with one participant’s data excluded because his/her score on the National Board of Medical Examiners (NBME) Comprehensive Basic Science examination (CBSE) was more than three standard deviations from the mean and was an extreme outlier. Out of the remaining 81 participants, 50.6% (41/81) were female with a mean age of 25.1 and 25.9% (21/81) having received only A letter grades during their first year of medical school. The survey revealed that 30.9% (25/81) of the students reported receiving a need-based scholarship. On average, the students studied 39.5 days, and all the students reported that First Aid for the United States Medical Licensing Examination (USMLE) Step 1 was their primary or secondary resource. UWorld Question Bank (UWorld) was cited as the other most common resource. The survey also revealed that 51.9% (42/81) of the students reported studying in a group at least part of the time. The mean Step 1 score for the participants was 240.1 (237.6 to 242.44, CI=95%), which was comparable to the two classes Step 1 means (238 and 239) provided to the school by USMLE. Most of the students reported spending “just the right amount” of time (52/81, 64.2%), with only 27.2% (22/81) of the students reporting that they did not spend enough time studying (*P*=0.002).

**Figure 1:**
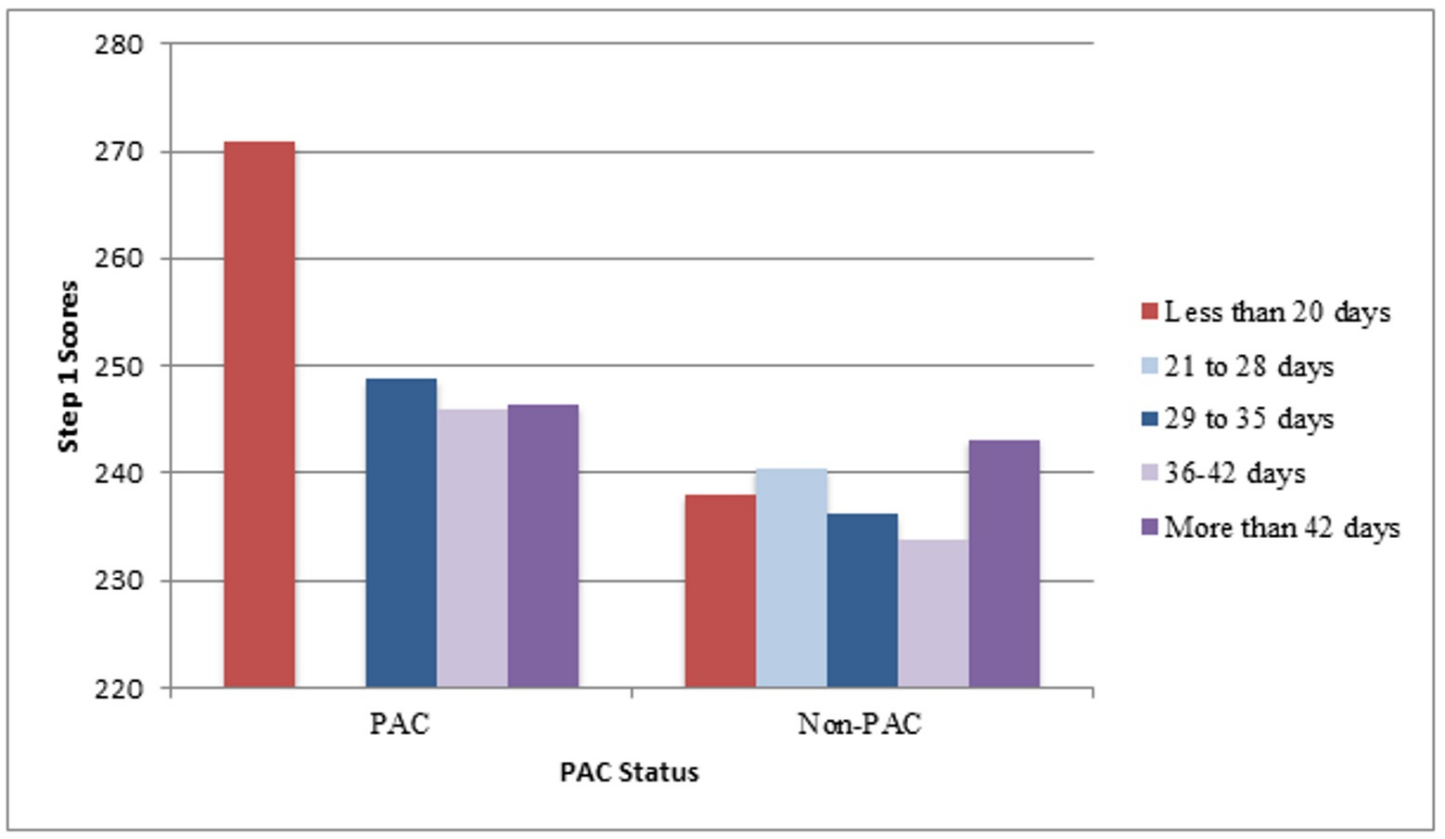
Comparison of Mean Step Scores for PACs vs. Non-PACs by Total Days Studied ^Abbreviations: PAC= Peer Academic Coach, one who received straight As during the first year of medical school^

Step 1 scores correlated well to the lack of financial need based on scholarship awarded (r=0.29, *P*=0.01), CBSE results (r=0.72, *P*<0.001), performance on UWorld (r=0.62, *P*<0.001), achieving straight As during first year (r=0.36, *P*=0.001), and for peer academic coaches (PACs), how many hours a PAC tutored (r=0.43, *P*<0.001). Step 1 scores did not correlate to age, gender, Medical College Admissions Test (MCAT) score, number of days studied, or perception of preparedness. Table 1 shows the means and correlations for some of these variables. Interestingly, there was a statistically significant difference in Step 1 scores between those receiving a financial need-based scholarship versus those who were not receiving such a scholarship (234.9 vs. 243.0, *P*=0.01). In addition, a statistically significant difference in Step 1 scores was detected between those who were PACs versus those who were not (248.6 vs. 237.7, *P*=0.001). Further, there was also a statistically significant difference in scores for PACs who worked one to ten hours per month and those who worked 11-20 hours per month (248.8 vs. 252.0, *P*=0.003). While there was no statistically significant difference in Step 1 scores among those who studied less than 20 days, 21–28 days, 29–35 days, 36–42 days, and greater than 42 days, there was a trend of increasing scores for those who had not obtained straight As to achieve near-PAC level performance if they had studied greater than 42 days (*P*=0.34) as can be seen clearly in Figure 1. Of note, only two students—one a PAC and one a non-PAC—studied less than 20 days and only two students, both non-PACs, studied between 21 and 28 days.

**Table 1:** Means and Correlation Coefficients of Variables Bolded Items are those in which the P value was statistically significant. Abbreviations: MCAT = Medical College Admission Test, CBSE = Clinical Basic Science Examination, USMLE = United States Medical Licensing Examination

Characteristic	Mean (95% CI, SD)	Pearson’s Correlation Coefficient to Step 1 Score (r)	*P* value
Age	25.1 (22 to 36, 2.3)	0.00	0.481
MCAT	30.8 (27 to 37, 2.2)	0.18	0.066
CBSE	198.9 (165 to 257, 18.6)	0.71	<0.001
Days Studied	39.4 (16 to 75, 9.5)	0.02	0.782
UWorld (%)	74.23 (45 to 94, 9.2)	0.62	<0.001
USMLE Step 1	240.5 (202 to 271, 13.2)	--	--

Lastly, we performed multiple linear regression in order to develop a predictive model utilizing the factors seen in Table 2. The unstandardized beta weights for the CBSE, students with straight As, financial need, and UWorld performance (in percentage) were 0.32, -3.82, 5.85, and 0.45. In other words, for every point of increased performance on the CBSE, the students could expect to have a 0.32 increase in their Step 1 scores, and for every percentage point improvement on UWorld, the students could expect to have a 0.45 increase in their Step 1 score. The interpretation of the beta coefficients for students with straight As and financial need may be confusing because of how these were coded. The students who did not get straight As were given a “2” and thus, not receiving straight As results in a 3.82 reduction in points. Likewise, the students not receiving financial aid were given a "2" and thus receive an additional 5.85 points on Step 1, in this model. Ultimately, 62.3% of variability can be accounted for by the following predictive model: 140.625+(0.319xCBSE)-(3.817xA)+(5.845xN)+(0.452xU) where A=1 if straight As and 2 if no straight As and where N=1 if receiving need-based scholarship and 2 if not, U=UWorld percentage and CBSE=the three-digit translated score of the CBSE taken prior to the dedicated study period. In terms of the contributions of each variable to this model, 97% of the financial need contribution is unique, 90.4% of the PAC is not dependent on the other independent variables, 66.2% of the CBSE is not dependent on the other independent variables, and 69.8% of the UWorld is not dependent on the other independent variables.

**Table 2:** Unstandardized Beta Weights and Unique Contributions of Independent Variables CBSE = Comprehensive Basic Science Examination

	Unstandardized Beta Weight	Unique Contribution (%)
CBSE	0.32	66.2
Straight As	-3.82	90.4
Financial Need	5.85	97
UWorld Performance	0.45	69.8

## Discussion

This study was the first in current literature to examine how students are preparing for the United States Medical Licensing Examination (USMLE) Step 1 examination. The top two resources were, not unsurprisingly, USMLE First Aid for Step 1 and UWorld Question Bank (UWorld). In our institution, the majority of students reported studying in groups for at least part of their study time. Finally, the students had a broad range of days studied—from less than three weeks to utilizing the entire dedicated study period. However, and perhaps most interestingly, Step 1 scores did not largely differ among those studying more or less than the mean study days. One potential explanation for this is that the students who performed well during the first two years of the curriculum self-selected to take the exam earlier whereas those who struggled may have decided to take more time. In fact, when we further examined the data to account for this, we found that for students who had done well during the first year of school, Step 1 performance did not improve with more days studied. However, for students who did not receive straight As during the first year of school, performance did improve with more days studied, almost to the mean of those who had straight As. This may offer hope to students who struggled during the first two years of the curriculum because putting in more study time for the Step 1 examination may help alleviate a potential disadvantage.

Not unsurprisingly, the students who performed the best on the exam were those students who performed well on the National Board of Medical Examiners (NBME) Comprehensive Basic Science Examination (CBSE), on UWorld, and during the first year of the curriculum. Of course, knowledge begets knowledge. However, what was perhaps surprising was that the students who had financial need did not perform as well as those who did not have financial need despite having access to the same preparation tools and curriculum, including free tutoring. It is possible that those with financial need had fewer resources during the actual curriculum such as supplemental books. The other possibility is that those with financial need may not have had the opportunity to take exam preparatory courses prior to medical school and thus may have not learned exam-taking skills. However, this still does not explain how they would still be able to do well on the CBSE and not as well on the actual exam.

There are several limitations to this study. First, the predictive model was developed from the experiences of one medical school and our overall sample size was small with a poor survey response rate. This may explain why other studies have been able to correlate MCAT scores to Step 1 scores while ours did not. This medical school's curriculum may be able to overcome some of the deficiencies seen in MCAT scoring and/or the students are provided with more test preparation skills and thus are able to overcome the lack of test-taking ability seen in their prior MCAT scores. In addition, there was a small difference in the mean Step 1 score of the class versus our sample (238.5 vs 240). It is unclear if this affected the overall results of our study. We were also unable to obtain data regarding the number of As received during the second year of the student curriculum and thus could not correlate scores to overall medical school performance in both first and second year.

## Conclusions

This study adds to the current knowledge of what may predict performance on the United States Medical Licensing Examination (USMLE) Step 1 exam by also studying how students at one institution currently prepare for the examination. Further, we have developed a predictive tool that may help students and mentors determine when the student is ready to take the Step 1 examination and succeed. Potential areas for future research include studying the link between financial need and performance on Step 1 as well as studying how utilizing different resources during the entire first- and second-year curriculum may influence the Step 1 exam score. Lastly, it would be ideal to validate this predictive model in other institutions. As obtaining a residency position becomes more and more competitive, research that helps students determine the best way to prepare for this exam will continue to be in demand.
